# Book Notices and Reviews

**Published:** 1864-06

**Authors:** 


### BOOK NOTICES AND REVIEWS.

MEDICAL DIAGNOSIS, WITH SPECIAL REFERENCE TO PRACTICAL MEDICINE.—*A Guide to the Knowledge and Discrimination of Diseases. By J. M. DaCosta, M. D., Lecturer on Clinical Medicine, and Physician to the Philadelphia Hospital; Fellow of the College of Physicians of Philadelphia; Member of the Pathological Society of Philadelphia; Corresponding Member of the New York Pathological Society, etc., etc. Illustrated with Engravings on Wood. Philadelphia. J. B. Lippincott & Co.* 1864.

In the preface the author states that his aim in writing this work has been to furnish advanced students and young graduates of medicines with a guide that might be of service to them in their endeavors to discriminate disease. And it will be readily conceded that every new fact which facilitates the discrimination of diseases, is an acquisition to medical science; that every appliance by which the signs or symptoms of disease are more distinctly pronounced; that every improvement in the arrangement or grouping of these signs and symptoms which render our diagnosis more ready and certain, are steps in the progress of improvement.

Great improvements have been made in the treatment of diseases within the last few years, and it will not be denied that the greater success of medical practice to-day, is due in no small degree, to a more accurate diagnosis, than it was possible to reach twenty years ago. The time has gone by when the physician can limit his means of diagnosis to the exercise of the special senses, vision, touch, etc.

Formerly we could go no further than these senses unassisted would carry us. But science has lent its aid, and furnished means by the help of which we can detect clearly what before we could not detect at all, or of what at best we only caught a glimpse. We now possess instruments by which we ascertain with accuracy the size of organs and their play. With thermometers we tell the heat of various parts of the body to a fraction of a degree. Specific-gravity bottles, and other measures devised for the purpose, inform us of the relative gravity of fluids. The microscope gives at a glance insight into matters far beyond anything the naked eye discloses. And chemistry, with its marvelous teachings, is rendering our knowledge of many morbid states admirably and amazingly complete. Then, too, the sagacity of modern times has taught us how to enlist the sense of hearing, and demonstrated how a disciplined ear may detect almost infallibly the workings of disease in cavities into which the eye can not penetrate. The effect of all these improved methods of study has been to give an immense impetus to clinical research, and in this manner to construct a solid groundwork of experience in striking contrast to the looseness and wild vagaries of former times. The advance in diagnosis thus attained forms indeed one of the most pleasing portions of medical history.

When, by means of the aided or unaided senses, the symptoms of the malady have been discovered, the next step toward a diagnosis is a proper appreciation of their significance and of their relation toward each other. Knowledge, and, above all, the exercise of the reasoning faculties, are now indispensable. The daily habit of investigating disease; an active pursuit of the anatomical lesions; chemistry, with its most searching analyses; the microscope, with all the wonders it reveals,—are all of little use, unless they have taught the necessity of placing the morbid signs they lay bare in connection with each other, and of considering in individual cases their respective value. Were it otherwise, the science of diagnosis would be simply a matter of memory. It is, however, this very analysis of symptoms, and the lengthy process of induction attending it, that makes medical diagnosis so difficult and so unattractive to the beginner.

Now, to accomplish all this effectually, the physician has need of much and varied knowledge. He must be master of something more than of that information supplied to him at hand by semeiology. He must be an anatomist to pronounce on the seat of the malady. He must be a physiologist to appreciate the aberration of functions. Above all, he must be a pathologist in the full sense of the term. He must understand the antagonism between diseases; the frequency with which they coexist; the influence of remedial agents on them. He must be cognizant of their natural history and of the general laws governing them—for how else can he form an estimate of morbid action while it is in progress? Then, too, it is desirable that he should be aware of what are their current divisions and classifications.

The author has given us, in this book, a clinical classification of the manifold and ever-varying features of disease, which, while it could be produced only by great labor, enhances immensely its value as a guide in practice. The plan followed is sufficiently indicated in the following brief analysis of the table of contents, viz., After the introductory general considerations,

Chapter I. Treats of the Examination of Patients, and some symptoms of general import.
" II. " " Diseases of the Brain, Spinal Cord, and their Nerves.
" III. " " Diseases of the Upper Air Passages.
" IV. " " " " Chest.
" V. " " " " Mouth, Pharynx, and Œsophagus.
" VI. " " Diseases of the Abdomen.
" VII. " " the Urine, and Diseases of the Urinary Organs.
" VIII. " " Dropsy.
" IX. " " Diseases of the Blood.
" X. " " Rheumatism and Gout.
" XI. " " Fevers.
" XII. " " Diseases of the Skin.
" XIII. " " Poisons and Parasites.

We have no hesitation in saying that the author in producing this work has conferred a real benefit upon the profession, and that no medical library is complete which does not contain a copy.

———

*A Treatise on Pharmacy*, Designed as a Text-Book for Students, and as a Guide for Physicians and Pharmaceutists, Containing the Officinal and many Unofficinal Formulas, and numerous examples of Extemporaneous Prescriptions. By Edward Parish. Third Edition, Thoroughly revised and improved, with important additions. With two hundred and thirty illustrations.

The present edition of this standard and popular work is greatly improved upon the last, is more ample, and is brought fully up to the present status of the science of art and pharmacy, and contains the working formulas for the old and new remedies recognized by the last edition of the Pharmacopœia, as well as many that are not officinal. From the title of the work, physicians might infer that it would not be of great value except in pharmacy, but this is an error, for many portions of it are as valuable to the physician in prescribing and administering medicine, as others are to the practical pharmaceutist. Especially will the practitioner be interested and instructed by the chapters on prescriptions, and on the art of selecting and combining medicines

It contains, too, a large number of extemporaneous combinations of medicines, arranged according to their proper classifications. In short, it is a book that no person engaged in the practice of pharmacy or medicine, should fail to be familiar with. It is a work of more than 800 pages, and being issued by Blanchard & Lea, of Philadelphia, the execution of it is unexceptionable.

